# 60-S Retrogated Compressed Sensing 2D Cine of the Heart: Sharper Borders and Accurate Quantification

**DOI:** 10.3390/jcm10112417

**Published:** 2021-05-29

**Authors:** Benjamin Longère, Christos V. Gkizas, Augustin Coisne, Lucas Grenier, Valentina Silvestri, Julien Pagniez, Arianna Simeone, Justin Hennicaux, Michaela Schmidt, Christoph Forman, Solenn Toupin, David Montaigne, François Pontana

**Affiliations:** 1 University of Lille, Inserm, CHU Lille, Institut Pasteur Lille, U1011—European Genomic Institute for Diabetes (EGID), F-59000 Lille, France; augustin.coisne@chru-lille.fr (A.C.); david.montaigne@chru-lille.fr (D.M.); francois.pontana@chru-lille.fr (F.P.); 2 CHU Lille, Department of Cardiovascular Radiology, F-59000 Lille, France; chgkizas@gmail.com (C.V.G.); lucas.grenier@chru-lille.fr (L.G.); valentina.silvestri@chru-lille.fr (V.S.); julien.pagniez@chru-lille.fr (J.P.); arianna.simeone@chru-lille.fr (A.S.); justin.hennicaux@chru-lille.fr (J.H.); 3 MR Product Innovation and Definition, Magnetic Resonance, Siemens Healthcare GmbH, 91052 Erlangen, Germany; michaela.schmidt@siemens-healthineers.com (M.S.); christoph.forman@siemens-healthineers.com (C.F.); 4 Scientific Partnerships, Siemens Healthcare France, 93200 Saint-Denis, France; solenn.toupin@siemens-healthineers.com

**Keywords:** cardiac, heart, magnetic resonance, CMR, compressed sensing, fast imaging, function, retrospective, retrogating, image quality

## Abstract

Background and objective: Real-time compressed sensing cine (CS_rt_) provides reliable quantification for both ventricles but may alter image quality. The aim of this study was to assess image quality and the accuracy of left (LV) and right ventricular (RV) volumes, ejection fraction and mass quantifications based on a retrogated segmented compressed sensing 2D cine sequence (CS_rg_). Methods: Thirty patients were enrolled. Each patient underwent the reference retrogated segmented steady-state free precession cine sequence (SSFP_ref_), the real-time CS_rt_ cine and the segmented retrogated prototype CS_rg_ sequence providing the same slices. Functional parameters quantification and image quality rating were performed on SSFP_ref_ and CS_rg_ images sets. The edge sharpness, which is an estimate of the edge spread function, was assessed for the three sequences. Results: The mean scan time was: SSFP_ref_ = 485.4 ± 83.3 (SD) s (95% CI: 454.3–516.5) and CS_rg_ = 58.3 ± 15.1 (SD) s (95% CI: 53.7–64.2) (*p* < 0.0001). CS_rg_ subjective image quality score (median: 4; range: 2–4) was higher than the one provided by CS_rt_ (median: 3; range: 2–4; *p* = 0.0008) and not different from SSFP_ref_ overall quality score (median: 4; range: 2–4; *p* = 0.31). CS_rg_ provided similar LV and RV functional parameters to those assessed with SSFP_ref_ (*p* > 0.05). Edge sharpness was significantly better with CS_rg_ (0.083 ± 0.013 (SD) pixel^−1^; 95% CI: 0.078–0.087) than with CS_rt_ (0.070 ± 0.011 (SD) pixel^−1^; 95% CI: 0.066–0.074; *p* = 0.0004) and not different from the reference technique (0.075 ± 0.016 (SD) pixel^−1^; 95% CI: 0.069–0.081; *p* = 0.0516). Conclusions: CS_rg_ cine provides in one minute an accurate quantification of LV and RV functional parameters without compromising subjective and objective image quality.

## 1. Introduction

Cardiac magnetic resonance (CMR) is the reference standard method for quantification of volumes, ejection fraction (EF) and mass of left (LV) and right ventricles (RV) [1,2,3]. Reliable volumes assessment is required since EF has a strong prognostic value regarding clinical outcomes and survival [4,5,6]. However, besides steady-state free precession cine images essential for quantification, phase contrast angiography, gadolinium enhanced imaging, and additional sequences may be recommended depending on heart conditions, leading to an extended scan time, which may be difficult to handle for patients suffering from cardiac-related shortness of breath, since multiple breath-holdings are required for the acquisition [7].

To reduce acquisition time, compressed sensing (CS) has recently been applied to magnetic resonance imaging, especially CMR [8]. Based on Candès et al.’s work on signal recovery from incomplete sampling, Donoho proposed the CS acquisition [9,10]. Since the signal compression required for archiving and transfer implies the deletion of most acquired data, the principle of CS is to acquire only the pieces of information that would be preserved after this compression, sparing the time necessary for the acquisition of the data that would finally be purged during this process. Three prerequisites are mandatory for CS [11]. First, contrary to most medical images, the signal must be compressible. This means a sparsifying transform is required for most transformed coefficients to be insignificant, making the transformed image compressible. Second, the undersampling must be incoherent to provide noise-like overfolding and avoid ambiguity. Finally, non-linear iterative reconstructions enforcing image consistency with measured signal and transformed image sparsity are needed. In the case of cine imaging, the redundancy in the cardiac cycle provides a strong spatiotemporal correlation, which can be exploited for additional acceleration [12,13,14]. Undersampling is not only performed in plane but also in the temporal domain by using variable sampling density maps for successive frames.

The first-generation real-time CS sequence (CS_rt_) has already been widely evaluated regarding volumes, EF and mass for both ventricles, at 1.5 and 3-Tesla [15,16,17,18,19,20,21,22,23,24,25]. These studies used 80 iterations to reconstruct CS_rt_ data, this setting being supposed to provide the best compromise between image quality and reconstruction time [17]. Nevertheless, computing times over one minute per slice were reported, making such a setting not compatible with clinical use [26,27]. Vermersch et al. evaluated the same sequence halving the number of iterations and demonstrated a similar agreement for the quantification of volumes, EF and mass quantification than previously reported in the literature, extending the use of CS_rt_ in clinical practice [22]. To provide an acceleration factor compatible with single-breath-hold real-time imaging, additional sensitivity encoding imaging (SENSE) and partial Fourier are implemented, the latter being a potential cause of edge sharpness impairment [28]. Another limitation is the limited temporal resolution of 49 ms which may provide variable numbers of frames per slice in the case of heart rate variability. To allow post-processing, a 25-frame per slice interpolation is performed. Besides the border blurring induced by this normalization, end-systolic volumes may be overestimated in the case of a fast heart rate [29,30].

A new generation of retrogated CS sequence (CS_rg_) has been released. It features segmented acquisition to improve temporal resolution and partial Fourier switch-off to improve edge sharpness. The purpose of this study was to assess the image quality and the ventricular functional parameters in comparison with CS_rt_ and the reference retrogated segmented steady-state free precession cine sequence (SSFP_ref_).

## 2. Materials and Methods

### 2.1. Study Population

From March to April 2019, 30 consecutive adult patients referred for rest CMR were included. Exclusion criteria were grown-up congenital heart disease work-up or follow-up, underaged patients, patients suffering from arrhythmia for whom the use of prospective ECG gating was necessary, MRI contraindications and patient refusal. Patients gave informed consent, and the protocol was approved by our Institutional Ethics Committee.

### 2.2. Imaging Protocol

CMR studies were performed on a 1.5-T scanner (MAGNETOM Aera, Siemens Healthcare, Erlangen, Germany). Every patient underwent three series of cine images: first the reference retrogated segmented multi-breath-hold SSFP sequence (SSFP_ref_); then the CS-accelerated SSFP real-time sequence (CS_rt_) acquired in two breath-holds, and finally the retrogated segmented SSFP prototype with CS-fashioned acceleration requiring three breath-holds (CS_rg_). One LV 2-chamber, one RV 2-chamber, one 4-chamber slice and a LV short-axis stack covering the entire ventricles were acquired with the three above-cited sequences, providing identical slice position, thickness, and number. Imaging parameters for the three sequences are summarized in Table 1.

### 2.3. Cine Images Quality Assessment

A 3-step evaluation was performed for the three sequences.

First, a subjective overall image quality was assessed using a 4-point Likert scale (1: non diagnostic, 2: fair, 3: good, 4: excellent).

Then acquisition quality was evaluated using a standardized score based on the “*LV-function cine SSFP*” section of the criteria established from the European CMR registry, evaluating the artifact detection [15] (p. 3). This score was modified, removing its four last items since their score was systematically null, accordingly to our center practice (Table 2). This score increased with acquisition impairment.

**Figure 1:**
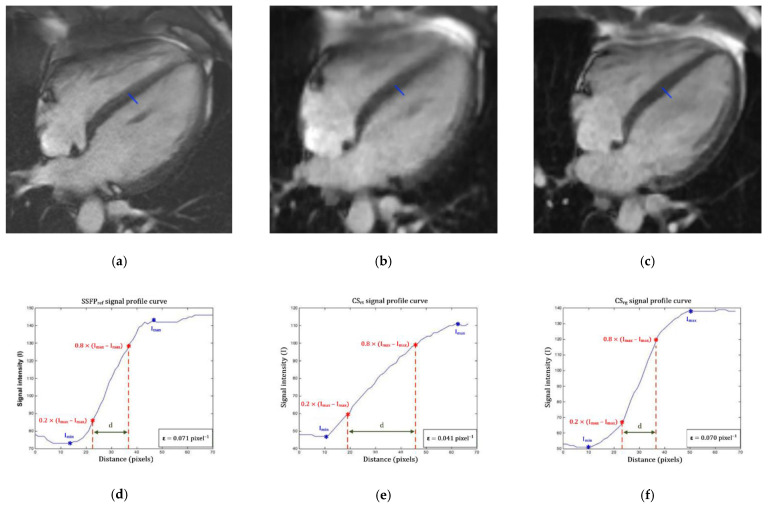
Edge sharpness measurement. An orthogonal profile line is drawn across the border between the interventricular myocardium and the left ventricular blood pool (blue line) on a 4-chamber view at end-diastole provided by (**a**) the reference segmented SSFP sequence, (**b**) the real-time compressed sensing sequence and (**c**) the retrogated compressed sensing prototype, providing intensity profiles (blue curves) along the line for (**d**) reference, (**e**) real-time and (**f**) prototype sequences. The edge sharpness was calculated as the inverse of the distance *d* (in pixels) from the positions corresponding to 20% and 80% (red stars) of the difference between the maximum and minimum signal intensities (black crosses) along the profile line and was expressed in pixel^−1^. Abbreviations: SSFP_ref_, reference steady-state free precession; CS_rt_, real-time compressed sensing; CS_rg_, retrogated compressed sensing; ε, edge sharpness; Ymax, maximum signal intensity; Ymin, minimum signal intensity; d, distance along the intensity (pixels).

Finally, the edge sharpness (ε) between myocardium and LV blood pool was measured on end-diastole 4-chamber view (Figure 1). This assessment was performed using a MATLAB (version R2015a, The MathWorks, Natick, MA, USA) homemade script. An intensity profile line was drawn perpendicularly to the mid-cavity interventricular septum border with the LV blood pool at end-diastole [31,32,33]. The ε value was a spatial frequency (pixel^−1^) calculated as the inverse of the distance separating the two points corresponding to 20% and 80% of the difference between the minimum and maximum intensities along this line.

### 2.4. Functional Evaluation

Assessment of end-diastolic volumes (EDV), end-systolic volumes (ESV), stroke volumes (SV) and EF were performed for both ventricles as well as LV mass (LVM). These parameters were measured on short-axis stacks with semi-automated segmentation with manual correction of the LV endocardium and epicardium while manual segmentation of the RV endocardium was necessary, using dedicated 4D analysis software (Cardiac MR analysis workflow, Syngo.via VB30A, Siemens Healthcare, Erlangen, Germany). Four-chamber, LV and RV 2-chamber slices were used to define mitral and tricuspid valve planes to ensure optimal assessment of ventricular bases.

### 2.5. Conditions of Image Analysis

The three datasets were independently analyzed by a 4-year experience CMR radiologist (LG). After anonymization and randomization performed for each sequence, each dataset was analyzed separately. Each analysis session, which evaluated all the images of the same sequence, was separated from the previous one by one month. A second radiologist with 11 years of experience in CMR (BL) segmented both ventricles on CS_rg_ images for interrater variability assessment.

### 2.6. Statistics Analysis

Categorical data were presented as numbers (percentage) and continuous variables as mean ± standard deviation (SD) (95% confidence interval (CI)) in the case of normal distribution or median (range: minimum–maximum) in other cases. Variable normality was assessed using the D’Agostino–Pearson test.

Paired Wilcoxon signed-rank test was used to compare subjective image qualities and acquisition qualities between CS_rg_ and SSFP_ref_ or CS_rt_. An analysis of variance was performed to compare edge sharpness and acquisition times of the three sequences. CS_rg_ and SSFP_ref_ mean functional parameters were compared using a Student’s *t* test, with linear regression and Bland–Altman analysis to assess the agreements between both methods. Inter and intra-observer variabilities were assessed using intra-class coefficient correlation. Significance of the test was defined by values of *p* < 0.05.

As for valvular regurgitations and WMD visualization, a receiver operating characteristic (ROC) curve was used. Statistical analysis was performed using dedicated commercially available software (MedCalc 14.8.1.0, MedCalc Software, Ostend, Belgium).

## 3. Results

### 3.1. Population Description

Demographics data are listed in Table 3. The 30 patients (22 men, 8 women; mean age: 48.0 ± 21.0 (SD) years; 95% CI: 40.2–55.9 years) were referred for: heart valve disease (*n* = 7/30; 23.3%), ischemic cardiopathy (*n* = 5/30; 16.7%); dilated cardiomyopathy (*n* = 5/30; 16.7%), myocarditis (*n* = 5/30; 16.7%), left ventricular hypertrophy (*n* = 5/30; 16.7%) and infiltrative cardiomyopathy (*n* = 3/30; 10%). All patients could fully complete CS_rt_ and CS_rg_ breath-holdings.

### 3.2. Scan Time and Image Quality

SSFP_ref_ mean scan time was 485.4 ± 83.3 (SD) s (95% CI: 454.3–516.5 s) while CS_rt_ scan time was 23.9 ± 5.2 (SD) s (95% CI: 21.9–25.8 s) and CS_rg_ scan time was 58.3 ± 15.1 (SD) s (95% CI: 53.7–64.2 s) (*p* < 0.0001). Compared to SSFP_ref_, the mean acceleration factor provided by CS_rg_ was 8.7 ± 2.6 (SD) (95% CI: 4.5–15.7). A mean number of 15.0 ± 1.2 (SD) slices (95% CI: 14.6–15.4 slices) was acquired with each technique. For CS_rg_ reconstruction of the full dataset, the mean time was 82.9 ± 23.4 (SD) s (95% CI: 77.4–110.1 s).

All images were rated as diagnostic. CS_rg_ subjective quality score (median: 4; range: 2–4) was higher than the one provided by CS_rt_ (median: 3; range: 2–4; *p* = 0.0008). SSFP_ref_ overall quality score (median: 4; range: 2–4) was not different from CS_rg_ score (*p* = 0.31).

Regarding the acquisition quality based on the EuroCMR registry, the CS_rg_ sequence (median: 0; range: 0–3) was not different from either the SSFP_ref_ acquisition (median: 0; range: 0–3; *p* = 0.38) or the CS_rt_ cine (median: 0; range: 0–3; *p* = 0.83).

**Figure 2:**
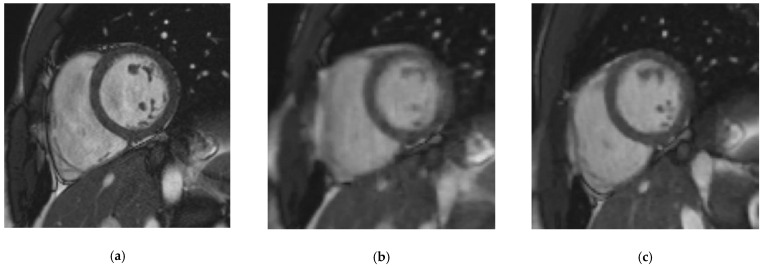
Midventricular short-axis cine slice acquired with the three cine sequences in a 37-year-old patient referred for myocarditis suspicion. (**a**) Reference steady-state free precession cine: Likert scale = 4/4; EuroCMR score = 0/10; ε = 0.082 pixel^−1^; LVEF = 59%, LVEDV = 135 mL, LVM = 83 g, RVEF = 63%, RVEDV = 159 mL; (**b**) Real-time compressed sensing cine: Likert scale = 4/3; EuroCMR score = 0/10; ε = 0.056 pixel^−1^; LVEF = 60%, LVEDV = 133 mL, LVM = 81 g, RVEF = 61%, RVEDV = 153 mL; (**c**) Retrogated compressed sensing cine: Likert scale = 4/4; EuroCMR score = 0/10; ε = 0.081 pixel^−1^; LVEF = 58%, LVEDV = 133 mL, LVM = 86 g, RVEF = 62%, RVEDV = 164 mL. Abbreviations: ε, edge sharpness; LVEF, left ventricular ejection fraction; LVEDV, left ventricular end-diastolic volume; LVM, left ventricular myocardial mass; RVEF, right ventricular ejection fraction; RVEDV, right ventricular end-diastolic volume.

**Figure 3:**
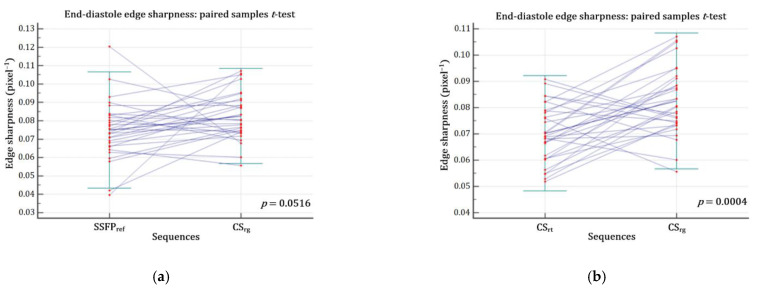
Edge sharpness comparison assessed at end-diastole. (**a**) There was no significant difference regarding ε between CS_rg_ and SSFP_ref_ (*p* = 0.0516) (**b**) but CS_rg_ significantly improved ε while compared with CS_rt_ (*p* = 0.0004). Abbreviations: CS_rg_, retrogated compressed sensing; CS_rt_, real-time compressed sensing; SSFP_ref_, reference steady-state free precession; ε, edge sharpness.

The CS_rg_ demonstrated a significantly better edge sharpness than CS_rt_ (ε_CSrg_ = 0.083 ± 0.013 (SD) pixel^−1^ (95% CI: 0.078–0.087 pixel^−1^) versus ε_CSrt_ = 0.070 ± 0.011 (SD) pixel^−1^ (95% CI: 0.066–0.074 pixel^−1^); *p* = 0.0004). Moreover, no significant difference was demonstrated between CS_rg_ and SSFP_ref_ (ε_SSFPref_ = 0.075 ± 0.016 (SD) pixel^−1^ (95% CI: 0.069–0.081 pixel^−1^); *p* = 0.0516) (Figure 2; Figure 3; Video S1; Video S2 (Supplementary Materials)).

### 3.3. Volumes, Functions and Mass Quantification

**Figure 4:**
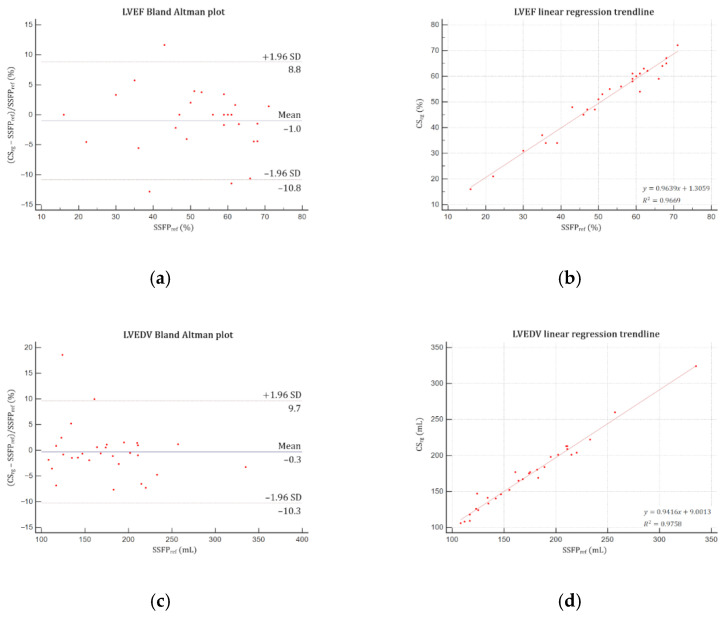

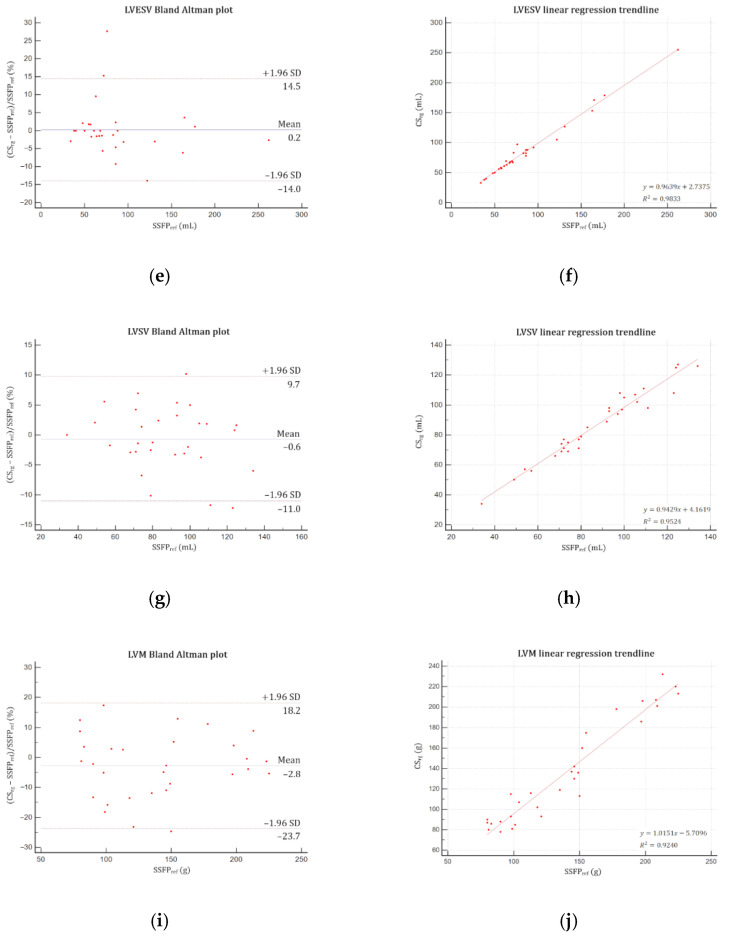
Bland–Altman plots and linear regression trendlines applied to left ventricular functional parameters quantifications. Left column: Bland–Altman plots for (**a**) LVEF, (**c**) LVEDV, (**e**) LVESV, (**g**) LVSV and (**i**) LVM. Solid blue lines are the mean differences between parameters measured with SSFP_ref_ and CS_rg_ sequences and dashed red lines are the 95% limits of agreement. Right column: linear regression trend lines for (**b**) LVEF, (**d**) LVEDV, (**f**) LVESV, (**h**) LVSV and (**j**) LVM. Abbreviations: SSFP_ref_, reference steady-state free precession; CS_rg_, retrogated compressed sensing; SD, standard deviation; LVEF, left ventricular ejection fraction; LVEDV, left ventricular end-diastolic volume; LVESV, left ventricular end-systolic volume; LVSV, left ventricular stroke volume; LVM, left ventricular mass.

**Figure 5:**
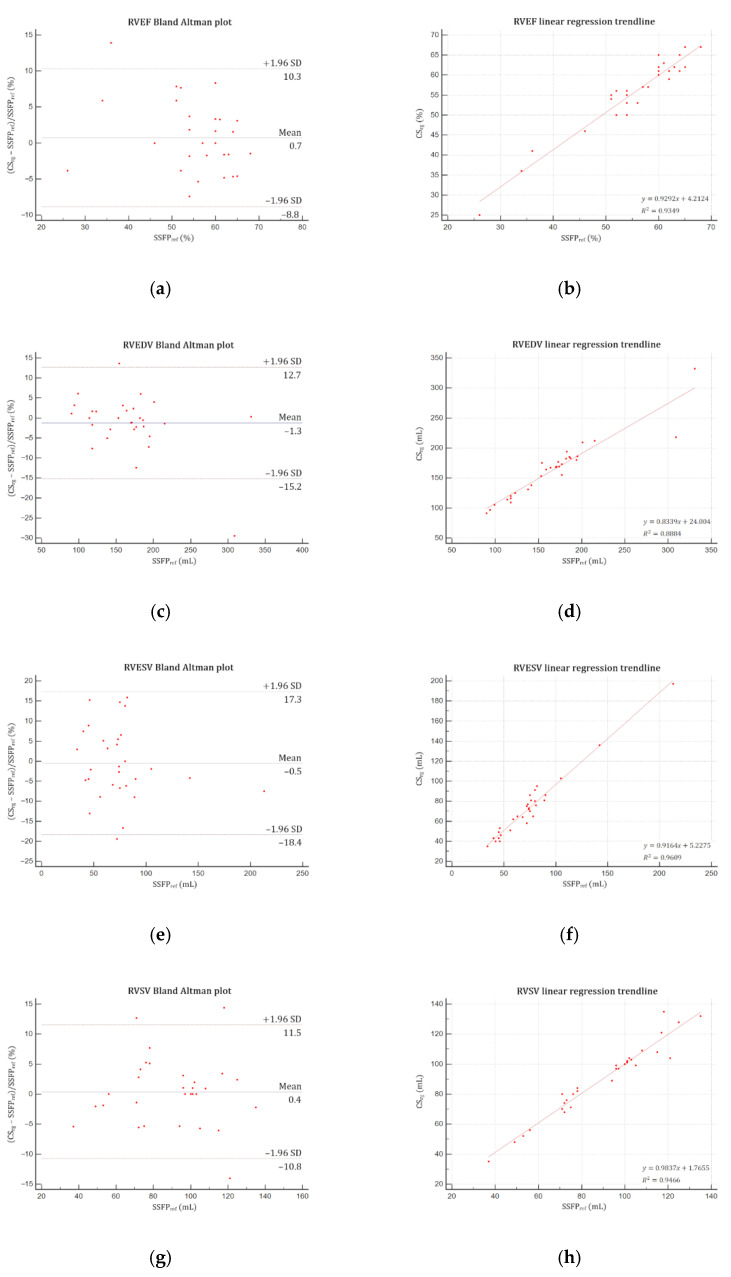
Bland–Altman plots and linear regression trendlines applied to right ventricular functional parameters quantifications. Left column: Bland-Altman plots for (**a**) RVEF, (**c**) RVEDV, (**e**) RVESV, and (**g**) RVSV. Solid blue lines are the mean differences between parameters measured with SSFP_ref_ and CS_rg_ sequences and dashed red lines are the 95% limits of agreement. Right column: linear regression trend lines for (**b**) RVEF, (**d**) RVEDV, (**f**) RVESV, and (**h**) RVSV. Abbreviations: SSFP_ref_, reference steady-state free precession; CS_rg_, retrogated compressed sensing; SD, standard deviation; RVEF, right ventricular ejection fraction; RVEDV, right ventricular end-diastolic volume; RVESV, right ventricular end-systolic volume; RVSV, right ventricular stroke volume.

Good agreements were yielded by Bland–Altman and linear regression analyses for both LV (Figure 4) and RV (Figure 5) assessments. No significant difference was demonstrated regarding LVM, LV and RV volumes (EDV, ESV, SV) and EF (*p* > 0.05) (Table 4). Intrarater variability was excellent, demonstrating intraclass correlation coefficients (ICC) greater than 0.99 for both ventricles, as were interrater variabilities for LV (ICC ≥ 0.97) and RV (ICC ≥ 0.96).

## 4. Discussion

Our clinical study performed a comprehensive evaluation of a retrogated CS sequence in daily practice. Results are in line with preliminary CS_rg_ tests on eight healthy volunteers reported by Forman et al., regarding LVEF, LVEDV and LVESV, which were similar to SSFP_ref_ quantification [34]. However, the study population was small and LVM, RV volumes and EF were not assessed. In the present study, no significant difference was demonstrated regarding LVM, LV and RV volumes (EDV, ESV and SV) and EF.

First-generation 49-ms temporal resolution CS_rt_ sequence provided LVEDV underestimation and LVM overestimation, which were clinically insignificant or smaller than intra or interrater variabilities [17,22,25,26,35]. Moreover, LVEDV underestimation was also reported with other acceleration techniques such as radial gradient-echo or k-space parallel imaging [36,37]. These differences were not depicted with CS_rg_. This might be explained by the improved edge sharpness facilitating segmentation, retrospective ECG gating allowing acquisition of the last phases of the cardiac cycle and the better temporal resolution (37 ms) [38]. This observation is in line with previous studies suggesting an optimal temporal resolution for accurate steady-state free precession quantification below 45 ms [29]. Since quantification is a major CMR point of interest, the absence of significant difference regarding each LV and RV parameter seems promising for clinical implementation [1]. Moreover, the high intra and interrater reproducibility allows the use of CS_rg_ for chronic status follow-up such as heart failure, anthracycline induced cardiotoxicity and other cardiomyopathies [39,40,41].

The overall image quality alteration using CS_rt_ has already been assessed in a previous publication [22]. The higher score provided by CS_rg_ confirmed the image quality improvement. Indeed, CS_rt_ interpolation was responsible for smoothed images, which required physicians to get used to this rendering. The absence of difference between acquisitions qualities (EuroCMR quality score) was expected. Indeed, no difference had been demonstrated regarding the first-generation CS sequence. The evaluated prototype features partial Fourier switch-off and segmented acquisition, which were not supposed to generate more artifacts. Regarding edge sharpness, the absence of interpolation and acquisition of more data improves boarder delineation. Not only was ε_CSrg_ better than ε_CSrg_ but it was similar to ε_SSFPref_. Edge sharpness was chosen as a metric for intrinsic image quality assessment due to its simple and reproducible implementation. Moreover, edge sharpness is an estimate of the edge spread function whose derivative is a line-spread function [33]. Fast Fourier transform of the latter gives the task-based modulation transfer function (MTF_Task_); edge sharpness can be considered as a reasonable approximation of MTF_Task_, which is widely used to evaluate the spatial frequency response of an imaging system, even using iterative reconstructions [42].

Predictably, CS_rg_ scan time was approximatively twice as long as the first-generation CS sequence since acquisitions were segmented on two heart beats in contrast to real-time imaging. However, as compared to SSFP_ref_, the mean CS_rg_ scan time of about 60 s still provides an 8.7-fold acceleration factor. The study protocol divided CS_rg_ acquisition in 3 stacks to shorten breath-holdings. Nevertheless, this setting is adjustable, such as the number of heart beats required for one slice to reduce apneas or increase spatiotemporal resolution. We chose to use CS to improve scan time, with workflow fluency being a major point of concern. However, it is possible to take advantage of this acceleration to improve spatial resolution, or maybe more interestingly, temporal resolution. Indeed, in the field of feature tracking in CMR, a resolution at least over 30 frames per cycle is recommended for accurate strain assessment, which is more time-consuming when using SSFP_ref_ [43]. Such settings may facilitate further feature tracking studies, though the impact of CS based reconstructions should be evaluated regarding strain analysis reliability.

The image quality improvement provided by the prototype CS_rg_ as compared to the first-generation real-time CS_rt_ should facilitate the spread of CS use in daily practice. However, the 2-shot acquisition is responsible for the loss of the real-time acquisition. Futures generations of CS cine sequences, maintaining the CS_rt_ real-time feature and the CS_rg_ image quality, should be oriented towards the application of additional motion correction algorithms to provide free-breathing acquisition with preserved image quality.

### Limitations

The size of the population was limited and cannot represent the whole variety of cardiac conditions encountered in daily practice. Nevertheless, the main objective of this study was to assess the image quality recovery as compared to SSFP_ref_ and CS_rt_. To facilitate the standardized assessment of edge sharpness, we chose to exclude congenital heart disease anatomy. Among the cardiac conditions assessed by CMR, dilated cardiomyopathy is a frequent pathology, usually responsible for shortness of breath, in which the LV wall may be thinned. The decreased wall thickness may be challenging for endocardium and epicardium delineation, thus impairing LV mass assessment. The impact of LV wall thickness for this assessment, for instance in dilated cardiomyopathy, could not be evaluated because of an insufficient subgroup. Nevertheless, we assume that the edge sharpness provided by CS_rg_ should help to distinguish the endocardium from the epicardium as compared to the first-generation CS_rt_ cine that provided blurrier borders. Although a high acceleration factor was demonstrated using CS_rg_, the improvement of CMR tolerance can only be assumed since this parameter was not evaluated. However, we suppose that the lower number of breath-holdings required by CS_rg_ should help dyspneic or claustrophobic patients undergo CMR examinations. This acceleration might be overestimated and must be interpreted cautiously since the two 1.6 × 1.6 mm^2^ in-plane resolution CS sequences were compared with a 1.3 × 1.3 mm^2^ in-plane resolution reference sequence. However, Miller et al. demonstrated that the maximal accuracy for functional parameters quantification using SSFP sequence was reached between 1 and 2-mm in-plane spatial resolution [29]. Even though further acquisitions could be performed during CS_rg_ reconstruction during this study (SSFP_ref_ images were acquired first and available), up to 2 min were necessary for images to be reconstructed and displayed to set the next sequences orientation in case of exclusive use of CS_rg_. Wall motion abnormalities were not evaluated, but the increased temporal resolution provided by segmented acquisition and the absence of interpolation should not impair their visualization on CS_rg_ since the first generation of compressed sensing cine was demonstrated to be reliable for this evaluation (Video S3 (Supplementary Materials)) [44]. The heart rate of the evaluated patients ranged from 54 to 101 beats per minute (R–R intervals: 594 ms to 1111 ms). Since CS_rg_ is a 2-shot sequence and its temporal resolution is constant (37 ms), the amount of data acquired during the acquisition must vary and may have an impact on the quality of the reconstructed cine images and consequently on functional assessment. The impact of heart rate on image quality was not evaluated in this study due to the limited size of the study population. Finally, only sinus rhythm patients were enrolled in this study. Even though a real-time acquisition is more robust than a segmented acquisition versus arrhythmia, the need of only two heart beats per slice should be more arrhythmia-proof than the conventional 8-heart-beat SSFP_ref_. Further comparison with other acceleration techniques such as generalized autocalibrating partial parallel acquisition would be interesting since the later requires more heart beats for identical temporal and spatial resolutions [45].

## 5. Conclusions

CS_rg_ allows reliable quantification of LV and RV volumes, EF and mass providing similar objective and subjective image quality to SSFP_ref_. Performed in clinical conditions, CS_rg_ is promising in terms of workflow improvement and image quality recovering in comparison with the first-generation real-time CS_rt_.

## Figures and Tables

**Table 1:** Imaging parameters of the reference steady-state free precession cine, real-time compressed sensing cine and segmented retrogated compressed sensing cine.

Parameters	SSFP_ref_	CS_rt_	CS_rg_
Repetition time—ms	3.16	2.70	2.70
Echo time—ms	1.23	1.14	1.14
Flip angle—degrees	57	60	60
Field of view—mm^2^	375 × 280	360 × 270	360 × 270
Matrix—pixels^2^	288 × 216	224 × 168	224 × 168
Spatial resolution—mm^2^	1.3 × 1.3	1.6 × 1.6	1.6 × 1.6
Temporal resolution—ms	37	49	37
Slice thickness/gap—mm	8/2	8/2	8/2
Bandwidth—Hz/pixel	915	900	900
Reconstructed cardiac phases—*n* Number of acquired cardiac phases	- 25 ^a^	25 ^a^ 16.8 ± 3.9	- 25 ^a^
Number of breath-holds	15.0 ± 1.2	2 ^a^	3 ^a^
Cycles per slice—*n*	8 ^a^	1 ^a^	2 ^a^
Cycles of iterative reconstruction—*n*	-	40	40

Data are expressed as mean ± standard deviation in the absence of any indication. ^a^ Constant value. Abbreviations: SSFP_ref_, reference steady-state free precession cine; CS_rt_, real-time compressed sensing cine; CS_rg_, segmented retrogated compressed sensing cine.

**Table 2:** “*LV-Function cine SSFP*” section of the standardized objective quality criteria score based on the European CMR registry. Adapted from [15] (p. 3).

Items	0	1	2	3	Maximum Score
**1. LV coverage**	**Full**	**-**	**No apex**	**Base or ≥1 slice missing**	**5**
**2. Wrap around**	**No**	**1 slice**	**2 slices**	**≥3 slices**	**3**
**3. Respiratory ghost**	**No**	**1 slice**	**2 slices**	**≥3 slices**	
**4. Cardiac ghost**	**No**	**1 slice**	**2 slices**	**≥3 slices**	
**5. Blurring/Mistriggering**	**No**	**1 slice**	**2 slices**	**≥3 slices**	
**6. Metallic artifacts**	**No**	**1 slice**	**2 slices**	**≥3 slices**	
**7. Shimming artifacts**	**No**	**1 slice**	**2 slices**	**≥3 slices**	
**8. Signal loss (coil inactive)**	**Activated**	**-**	**Not activated**		**2**
*9. Orientation of stack*	*Correct*	*-*	*Incorrect*	*-*	*2*
*10. Slice thickness*	*≤10 mm*	*11–15 mm*	*-*	*>15 mm*	*3*
*11. Gap*	*≤3 mm*	*3–4 mm*	*-*	*>4 mm*	*3*
*12. Correct LV long axes*	*≥2 mm*	*1*	*-*	*None*	*3*
*Score*					*21*
**Modified score (items 1 to 8)**					**10**

The four last items were nulled since acquisitions were repeated when orientation was not appropriated (item 9 = 0); all acquisitions were performed using a 8-mm thickness (item 10 score = 0) and a 2-mm gap (item 11 score = 0), horizontal and vertical long axis views were systematically acquired (item 12 score = 0). Consequently, italic criteria were not applied, and only bold criteria were used for objective quality assessment in our study, providing a maximum score of 10 points. The more artifacts there were, the higher the score was. Abbreviations: LV, left ventricle; SSFP, steady-state free precession.

**Table 3:** Study population characteristics.

	Mean ± SD (95% CI)	Minimum Value	Maximum Value
Age—years	48.0 ± 21.0 (40.2–55.9)	18	87
Weight—kg	73.9 ± 12.1 (69.4–78.5)	53	105
Height—cm	172.5 ± 8.3 (169.4–175.6)	157	189
Body surface area—m^2^	1.87 ± 0.17 (1.80–1.93)	1.55	2.22
Body mass index—kg/m^2^	24.8 ± 3.6 (23.5–26.2)	19.8	33.7
Heart rate—beats per minute	73.8 ± 13.5 (68.7–78.9)	54	101

Abbreviations: SD, standard deviation; 95% CI, 95% confidence interval.

**Table 4:** Functional parameters segmented on both the reference steady-state free-precession and retrogated compressed-sensing cine.

	SSFP_ref_ Sequence (Mean ± SD (95% CI))	CS_rg_ Sequence (Mean ± SD (95% CI))	Difference (Mean ± SD (95% CI))	Paired *t* Test *p*	ICC	
					Inter	Intra
LVEF—%	52.7 ± 14.1 (47.5–58.0)	52.1 ± 13.8 (47.0–57.3)	−0.6 ± 2.6 (−1.6 to −0.4)	0.21	0.99	0.9996
LVEDV—mL	174.1 ± 50.3 (155.3–192.9)	173.0 ± 48.0 (155.1–190.9)	−1.2 ± 8.0 (−4.2 to −1.8)	0.43	0.99	0.9994
LVESV—mL	86.8 ± 49.4 (68.4–105.2)	86.4 ± 48.0 (68.5–104.3)	−0.4 ± 6.5 (−2.8 to −2.0)	0.74	0.98	0.9906
LVSV—mL	87.5 ± 24.0 (78.6–96.5)	86.7 ± 23.2 (78.0–95.4)	−0.8 ± 5.3 (−2.8 to −1.1)	0.39	0.98	0.9932
LVM—g	139.5 ± 47.7 (121.6–157.3)	135.9 ± 50.4 (117.0–154.7)	−3.6 ± 13.9 (−8.8 to 1.6)	0.17	0.97	0.9994
RVEF—%	55.8 ± 9.7 (52.2–59.4)	56.0 ± 9.3 (52.6–59.5)	0.3 ± 2.4 (−0.7 to 1.2)	0.56	0.96	0.9965
RVEDV—mL	167.0 ± 53.6 (147.0–187.0)	163.2 ± 47.4 (145.5–180.9)	−3.8 ± 18.2 (−10.5 to 3.05)	0.27	0.98	0.9968
RVESV—mL	74.1 ± 34.3 (61.3–86.9)	73.1 ± 32.0 (61.1–85.0)	−1.0 ± 6.9 (−3.6 to 1.6)	0.45	0.96	0.9934
RVSV—mL	89.8 ± 24.3 (80.8–98.9)	90.1 ± 24.5 (81.0–99.3)	−0.3 ± 5.7 (−1.8 to 2.4)	0.77	0.97	0.9961

ICC assessed the inter and intrarater agreements for ventricular segmentations. The significance of Student’s *t* test is defined by values of *p* < 0.05. Abbreviations: SSFP_ref_, reference steady-state free-precession cine; CS_rg_, retrogated compressed-sensing cine; SD, standard deviation; 95%CI, 95% confidence interval; LV, left ventricular; RV, right ventricular; EF, ejection fraction; EDV, end-diastolic volume; ESV, end-systolic volume; SV, stroke volume; LVM, left ventricular mass; ICC, intraclass correlation coefficient; Inter, interrater; Intra, intrarater.

## Data Availability

The data presented in this study are available on reasonable request from the corresponding author, subject to approval by the research ethics committee of Lille University Hospital.

## Supplementary Materials

The following are available online at https://www.mdpi.com/article/10.3390/jcm10112417/s1, Video S1: Short-axis cine stacks in a 45-year-old woman referred for myocarditis follow-up; Video S2: Four-chamber cine slice in the same patient as Video S1; Video S3, short-axis mid-cavity cine slice in a 68-year-old patient referred for viability assessment after myocardial infarction, demonstrating inferolateral akinesia.
